# Construction of Photoinitiator Functionalized Spherical Nanoparticles Enabling Favorable Photoinitiating Activity and Migration Resistance for 3D Printing

**DOI:** 10.3390/polym14214551

**Published:** 2022-10-27

**Authors:** Lijuan Chen, Fan Chen, Weixin Zhu, Luoyi Chen, Yingyu Liang, Xiaohui Guo

**Affiliations:** 1 Guangzhou Key Laboratory of Sensing Materials & Devices, Center for Advanced Analytical Science, School of Chemistry and Chemical Engineering, Guangzhou University, Guangzhou 510006, China; 2 School of Chemistry and Chemical Engineering, Qiannan Normal University for Nationalities, Duyun 558000, China

**Keywords:** multifunctional photoinitiator, 3D printing, UV-curing polymer, nanomaterial, immobilization

## Abstract

A straight-forward method was exploited to construct a multifunctional hybrid photoinitiator by supporting 2-hydroxy-2-methylpropiophenone (HMPP) onto a nano-silica surface through a chemical reaction between silica and HMPP by using (3-isocyanatopropyl)-triethoxysilane (IPTS) as a bridge, and this was noted as silica-s-HMPP. The novel hybrid-photoinitiator can not only initiate the photopolymerization but also prominently improve the dispersion of nanoparticles in the polyurethane acrylate matrix and enhance the filler-elastomer interfacial interaction, which results in excellent mechanical properties of UV-cured nanocomposites. Furthermore, the amount of extractable residual photoinitiators in the UV-cured system of silica-s-HPMM shows a significant decrease compared with the original HPMM system. Since endowing the silica nanoparticle with photo-initiated performance and fairly lower mobility, it may lead to a reduction in environmental contamination compared to traditional photoinitators. In addition, the hybrid-photoinitiator gives rise to an accurate resolution object with a complex construction and favorable surface morphology, indicating that multifunctional nanosilica particles can be applied in stereolithographic 3D printing.

## 1. Introduction

Photo-induced free radical polymerization is widely used in conventional applications and high-tech domains such as coatings, adhesives, photoresists, 3D printing, and dental materials due to its fast curing speed, low energy consumption, and low environmental pollution [1,2,3,4,5]. Among photoinitiators for free radical polymerization, *α*-hydroxyalkylphenone photoinitiators (PIs), for example, HMPP, HHMP, and HCPK, have high photoactivity, high thermal stability, low odor, good solubility, and great coloring property in clear coatings [6,7]. Furthermore, there are many researchers investigating the physicochemical and photophysical characteristics of α-hydroxyalkylphenone PIs [8,9]. However, there is a critical problem that the photolysis products or undecomposed molecules of the small molecular photoinitiator may migrate from the cured matrix to the surface or be released to the outside, which seriously affects its applicability to some specific areas, such as food packaging and biomaterials [10,11,12].

Nowadays, to overcome the migration of low molecular photoinitiators, there have been increasing efforts towards the synthesis of higher molecular, polymerizable, or oligomeric photoinitiators [13,14,15,16], but less effort focused on hybrid organic-inorganic photoinitiators [17,18]. Hybrid photoinitiators are composites containing both inorganic and organic ingredients linked by stable covalent bonds, which also actually achieve increased molecular weight via decorating the photoinitiator on the nanoparticle surface. The grafting of photoinitiators onto the surfaces of different inorganic fillers such as montmorillonite, zirconium, and titanium dioxide has been reported in literature [19]. Melinte et al. presented [20] a collection of high-performance hybrid nanocomposite coatings with photoinitiator-modified montmorillonite. Zhou et al. prepared [21] the multi-functional groups zirconium propoxide-based photoinitiators through ligand substitution. Furthermore, inorganic nanoparticles are widely used in polymer-based composites to improve the heat resistance, mechanical properties, and transmittance because of their excellent thermal properties, low thermal expansion, high modulus, and so on [22,23,24,25,26]. However, due to the high surface activity of the pristine nanoparticles, they tend to agglomerate with inferior dispersion. During the past decades, many studies have demonstrated improved filler dispersion in the matrix, which commonly adopts a simple method such as surface modification with silane coupling [27,28,29,30,31]. Hence, after endowing the inorganic nanoparticles with photo-initiated characteristics, it can provide a new perspective for the obtainment of UV-cured composites with excellent performance.

In this study, free radical type photoinitiator HMPP was anchored on a silica surface based on a silane coupling agent (IPTS) as a bridge, and noted as silica-s-HMPP. The hybrid organic-inorganic photoinitiator silica-s-HMPP was introduced to the urethane acrylate-based elastomer for the production of a higher performance UV resin for SLA 3D printing. The UV absorption capacity and thermal stability were investigated by FT-IR, UV-vis spectroscopy, and TGA. To realize the practical benefit of the hybrid photoinitiator, it was also applied as a kind of modified photoactive filler mixed into polyurethane acrylate. The performance of PUA/silica-s-HMPP systems such as kinetics of photopolymerization, mechanical property, and migration property was also studied and compared with commercial HMPP and the physical mixture of HMPP with silica. The appropriate amount of silica-s-HMPP in PUA resin can result in complex-structured 3D printed objects with high surface quality and excellent mechanical performance.

## 2. Materials and Methods

### 2.1. Materials

Precipitated silica (particle diameter of 10–20 nm and a surface area of 200~220 m^2^/g) was purchased from Huiming Chemical Co., Ltd. (Yichun, Jiangxi, China). 2-Hydroxy-2-methylpropiophenone (HMPP) was obtained from Aladdin Reagent Co. (Shanghai, China). (3-isocyanatopropyl)-triethoxysilane (IPTS) was purchased from Nanjing Pinning Coupling Agent Co., Ltd. (Nanjing, China). Urethane acrylate (ROYJI RJ-425, M_n_ = 500 and RJ-426, M_n_ = 2800), the two oligomers and 4-acryloylmorpholine (ACMO) were obtained from Guangzhou Lihou Trading Co., Ltd. (Guangzhou, China). Absolute ethyl alcohol and other reagents used without further purification were obtained from the Guangzhou Chemical Reagent Factory (Guangzhou, China).

### 2.2. Preparation of Silica-s-HMPP

**Figure 1:**
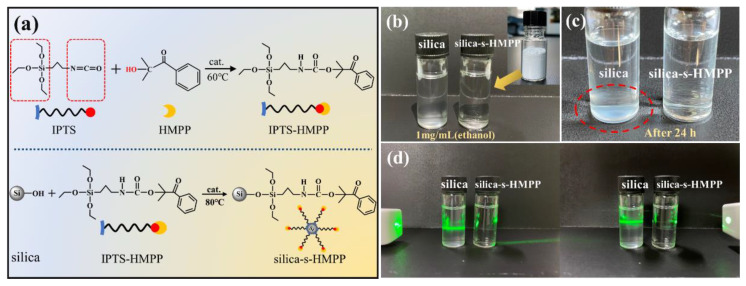
(**a**) Illustration of the hybrid photoinitiator silica-s-HMPP preparation (**b**) photos of original state of the silica and silica-s-HMPP dispersing in ethanol (1 mg/mL) and (**c**) standing for 24 h, (**d**) the photos of silica and silica-s-HMPP ethanol solution under a laser irradiation, respectively.

The hybrid photoinitiator silica-s-HMPP was prepared according to Figure 1a. Firstly, 6.17 g (0.025 mol) of IPTS, 4.1 g (0.025 mol) of HMPP, and 0.03 g of dibutyltin dilaurate were gradually added into a three-necked flask and stirred at room temperature for 10 min. Then, the reaction was carried out for 3 h at 60 °C in a nitrogenous atmosphere. The intermediate product (IPTS-HMPP) was transferred to a one-necked flask for sealing without aftertreatment. To achieve silica-s-HMMP, 10 g of SiO_2_ was placed in a 500 mL three-necked flask, then 4 g of IPTS-HMPP was added dropwise into the flask with intense agitation for 3 h at 80 °C. Finally, the product was washed with 300 mL absolute ethyl alcohol 3 times, and after drying at 60 °C to constant weight, the hybrid photoinitiator (silica-s-HMPP) was obtained.

### 2.3. Preparation of the Hybrid Materials

The hybrid materials were prepared via photopolymerization with different kinds of photoinitiators, such as HMPP and silica-s-HMPP. The formulas of the UV-cured hybrid materials are listed in Table 1. The content of silica and HMPP was determined by the residues of thermogravimetric analysis (TGA) to ensure accurate contents of photoinitiator and filler in each hybrid material. Each sample was dispersed ultrasonically for 30 min until the hybrid system was mixed evenly. The homogeneous compositions were poured into a petri dish with a diameter of 75 mm and placed until the bubbles disappeared, and then exposed to UV irradiation for a period of 10 s by an Hg-Xe lamp with a light intensity of 80 mW/cm^2^.

### 2.4. Stereolithographic 3D Printing

**Scheme 1:**
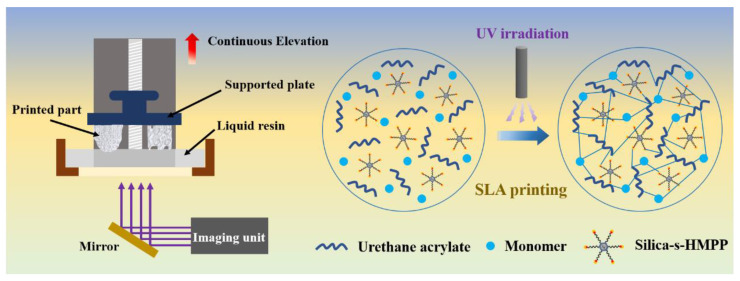
Schematic of the SLA printing process and the formation of the UV cured crosslinking hybrid network.

A desktop SLA 3D printer (Form 3, Formlabs, Somerville, MA, USA) was used to produce 3D prints from the mixed photocurable resin (300 mL, including PUA, diluent, and hybrid photoinitiator silica-s-HMPP) in the defined build volume (145 × 145 × 185 mm^3^). The laser source of the SLA 3D printer possessed a power of 0.25 W, a wavelength of 405 nm and a laser spot size of 85 μm. For 3D printing, the slice thickness was selected at 100 μm and the exposure time for each layer was 10 s. The 3D printing was conducted by raising the platform from the bottom up and with the laser light shifting back and forth under the resin tank. When the print program finished, all printed objects were fetched from the platform and then soaked in an auto-washing machine filled with absolute ethyl alcohol for 5 min to remove the uncured resin liquid. Final UV post treatment was carried out by exposing the printed object to a 365 nm UV irradiation for 5 min. The schematic of the SLA printing process and the formation of the UV cured crosslinking hybrid network is shown in Scheme 1.

### 2.5. Characterization

FT-IR spectrum was carried out on a Nicolet Magna 360 spectrometer and scanned from 4000 cm^−1^ to 400 cm^−1^. UV absorption spectra of samples were recorded on a PERSEE UV-1810 spectrophotometer. The concentration of each sample was 5 × 10^−5^ mol/L in C_2_H_5_OH. Thermal gravimetric analysis (TGA) was performed on a NETZSCH STA 499C in an N_2_ atmosphere from 30 °C to 800 °C using a heating rate of 10 °C/min. The photopolymerization kinetics was investigated by the-ThermoFisher Nicolet 6700 real-time FT-IR (Waltham, MA, USA) with a horizontal sample holder. A high-pressure mercury lamp, MEJIRO MUA-165, was directed at the samples during the test (Tokyo, Japan). The UV source was in the range of 250–365 nm. The double bond conversion (C_db_) was calculated from the variation of the absorption peak at 795–825 cm^−1^ for the acrylate double bond (C=C), and the characteristic absorption peak of the carbonyl group (C=O) at 1720 cm^−1^ was used as the internal standard. The C_db_ was calculated by Equation (1):

(1) $$C_{\mathrm{db}}/\%=\frac{{\left(A_{795\sim 825}{/A}_{1720}\right)}_{t}-{\left(A_{795\sim 825}{/A}_{1720}\right)}_{0}}{{\left(A_{795\sim 825}{/A}_{1720}\right)}_{0}}\times 100$$

where, ${\left(A_{795\sim 825}{/A}_{1720}\right)}_{0}$ and ${\left(A_{795\sim 825}{/A}_{1720}\right)}_{t}$ were the relative peak area ratio of C=C to C=O before and after UV irradiation curing at variational time (t), respectively. The samples for mechanical test were made by a dumbbell cutter from the UV cured composites. The tensile property was operated according to the ASTM D638 03, and for each sample at least five measurements were performed.

## 3. Results

### 3.1. Photoinitiator HMPP Supported onto Silica Surface

The photos of silica and silica-s-HMPP dispersing in ethanol, respectively, and placing of them at different time points are shown in Figure 1b,c. Silica nanoparticles precipitate after 24 h but the status of silica-s-HMPP has not changed, and there is variable as well as no obvious precipitation. Interestingly, the Tyndall effect of the silica ethanol solution has vanished after modification by HMPP. The solubility of silica-s-HMPP in ethanol solvent has been enhanced, and the colloidal solution of pristine silica nanoparticles has turned into a clear and transparent solution as shown in Figure 1d.

**Figure 2:**
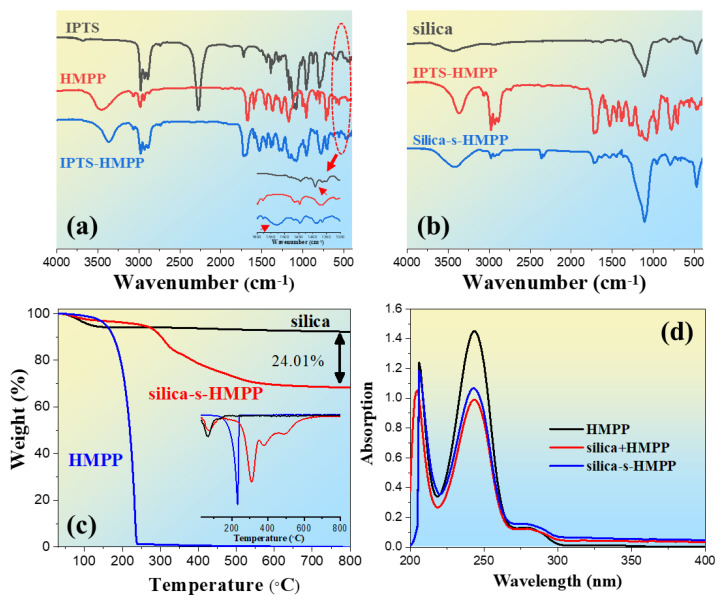
FTIR spectra (**a**) of IPTS, HMPP, and IPTS-HMPP and (**b**) of silica, IPTS-HMPP, and silica-s-HMPP; (**c**) TGA curves of silica, HMPP, and silica-s-HMPP (the insert curve is DTG curves), and (**d**) UV-vis absorption of HMPP, silica-s-HMPP, and the mixture of HMPP and silica (silica+HMPP).

Because of its excellent properties over pure constituents, the combination of organic and inorganic components has become a significant method in the development of novel materials. Figure 2a,b illustrates the FTIR spectra of pristine IPTS, HMPP, IPTS-HMPP, silica, and silica-s-HMPP, respectively. Compared to the spectrum of IPTS, the characteristic peak at 2279 cm^−1^ disappears in the spectrum of IPTS-HMPP, which is attributed to the asymmetric stretching vibration of -NCO. Furthermore, a new prominent peak around 1530 cm^−1^ attributed to a urethane bond appeared due to the reaction between IPTS and HMPP. For the spectrum of silica-s-HMPP, the -CH_2_ vibration at 2900 cm^−1^ and the C=O vibration at 1705 cm^−1^ are significantly observed. A large mutation occurred at the peak around 1000~1250 cm^−1^, which is attributed to the Si-O-Si vibration, implying successful grafting of the IPTS-HMPP onto the silica surface. 

TGA is performed to estimate the loading amount of IPTS-HMPP on the silica surface, as shown Figure 2c. The approximate loading amount of HMPP on the silica surface can be calculated according to the molar mass of IPTS and HMPP. To compare thermal stability, the derivative thermogravimetry analysis (DTG) of silica, HMPP, and silica-s-HMPP is illustrated as an insert curve and are shown in Figure 2c. Based on the weight loss of silica and silica-s-HMPP, the loading amount of IPTS HMPP on silica-s-HMPP surface is 24.01 wt.%. According to the literature [14], the graft ratio of HMPP is calculated as 9.59 wt.%, corresponding to 0.584 mmol HMPP per 1 g of silica-s-HMPP. As shown in the DTG curves, the thermal decomposition temperature of HMPP is 232 °C and of silica-s-HMPP is 310 °C, a 78 °C increase. The curve of neat silica has only one peak near 70 °C due to the decomposition of the water bond on the silica surface. Based on the covalent bond between silica and HMPP, the inorganic-organic photoinitiator silica-s-HMPP particles are more thermal stable than the traditional small-molecule photoinitiator HMPP. UV-vis spectra of HMPP, silica-s-HMPP, and the mixture of HMPP and silica (silica+HMPP) in absolute ethanol are illustrated in Figure 2d. The concentration of each sample is 1 × 10^−4^ mmol L^−1^ in terms of HMPP moieties. All the three samples exhibit the usual characteristic absorption spectra of benzophenone, which has characteristic absorptions at λ = 243 nm and 205 nm. However, silica-s-HMPP and the equal-content of silica and HMPP mixture possess weaker absorptions compared to HMPP, which shows that the silica nanoparticle has a nonnegligible influence on the UV-vis absorption of HMPP due to the non-transparent inorganic silica nanoparticles, and they generally give rise to scatter and reflect ultraviolet light [32].

### 3.2. Kinetic Study of Photopolymerization

**Figure 3:**
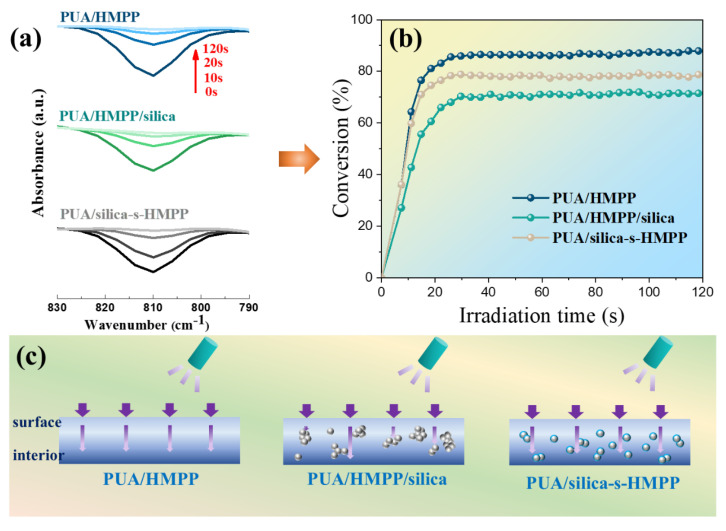
(**a**) Real-time FTIR curves of the photopolymerization of three samples, (**b**) Conversion vs. irradiation time plots for the UV-curable samples and (**c**) Illustration of the photopolymerization process of the three samples.

The photoactivity of silica-s-HMPP was assessed by using real-time FTIR to further compare the photoinitiating efficiency with different photoinitiators in a urethane acrylate matrix. The real-time FTIR curves of the photopolymerization of three samples are shown in Figure 3a. The conversion vs. irradiation time plots for the UV-curable samples are shown in Figure 3b. The polymerization behavior of the PUA/silica-s-HMPP system appears similar to the traditional free radicals that initiate photopolymerization, which means that the supported photoinitiator can effectively initiate polymerization of monomers or polymers under UV-light irradiation. Importantly, the addition of nanoparticles such as silica or silica-s-HMPP has mildly retarded the double bond conversion of the UV-systems due to the reduction of light absorption efficiency of the photoinitiator and hinders the UV curing behavior. However, after the silica surface is grafted with the photoinitiator, the phenomenon of low light absorption efficiency caused by nanoparticle blocking was improved due to the anchoring of the HMPP to the large specific surface of nanosilica. This allows for tridimensional space dispersion of the photoinitiator to improve the light absorption efficiency compared to that of the physical mixture system (PUA/HMPP/silica) as shown in Figure 3c. In addition, the conversion rate of double bonds has a great relationship with the fluidity of the hybrid UV-curing system. After adding silica or modified silica, the fluidity of photosensitive resin is affected, which results in poor fluidity of the PUA/HMPP/silica and the PUA/silica-s-HMPP systems. Furthermore, the final conversion of the PUA/HMPP system is 87.8% which is higher than that of the silica-filled system. It is mainly due to the newly formed urethane bond through the chemical reaction between IPTS and HMPP, which belongs to the electron withdrawing group, which can mildly impact the photo-initiating activity of the silica-s-HMPP. The lowest final conversion of the PUA/HMPP/silica system may be due to the lower photo-initiating activity and oxygen inhibition [33].

### 3.3. Mechanical Properties and Morphology of Polyurethane Acrylate Composites

**Figure 4:**
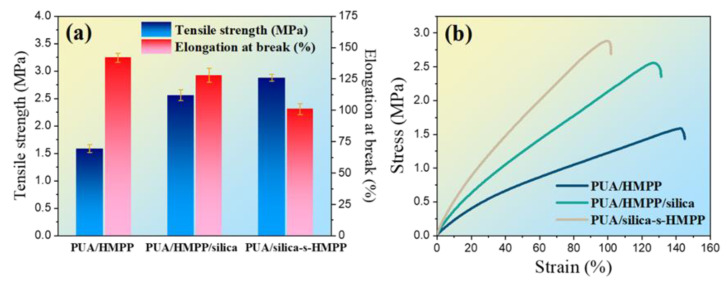
Mechanical properties of PUA composites: (**a**) tensile strength and elongation at break, (**b**) stress vs. strain curves of PUA composites.

The combining of organic and inorganic hybrid components has become a significant method in creating novel materials with remarkable properties superior to the use of a single ingredient alone. The use of these has been verified by many successful applications in nanoscience and nanotechnologies [34,35,36]. The mechanical properties of 3D printed urethane acrylate composites photoinitiated by different photoinitiators are compared in Figure 4. After adding rigid particles such as silica or silica-s-HMPP into the polyurethane acrylate matrix, which increases the tensile strength and elasticity of the modulus, there is a slight decrease in the elongation at break. The PUA/silica-s-HMPP system achieves approximately 81.1% and 12.5%, 127.9% and 47.7% increase in tensile strength and elasticity modulus, respectively, when compared to the PUA/HMPP and the PUA/HMPP/silica photoinitiated by HMPP as the photoinitiator, even though the elongation at break only slightly decreased. It is because of this increase in tensile strength and elasticity modulus through silica surface modified by IPTS-HMPP can lead to a remarkable improvement in the interfacial interaction between inorganic nanoparticles and UV resin matrices, ultimately resulting in the excellent mechanical properties. Besides, the elongation break of PUA/silica-s-HMPP, the photopolymerization system is lower in PUA/silica-s-HMPP than in the other systems due to the improved interfacial rigid deformation, indicating the existence of a rigid interface layer between silica and the elastomer matrix [37].

### 3.4. Migration and Mechanism of the Photoinitiator in the UV-Cured Composites

**Figure 5:**
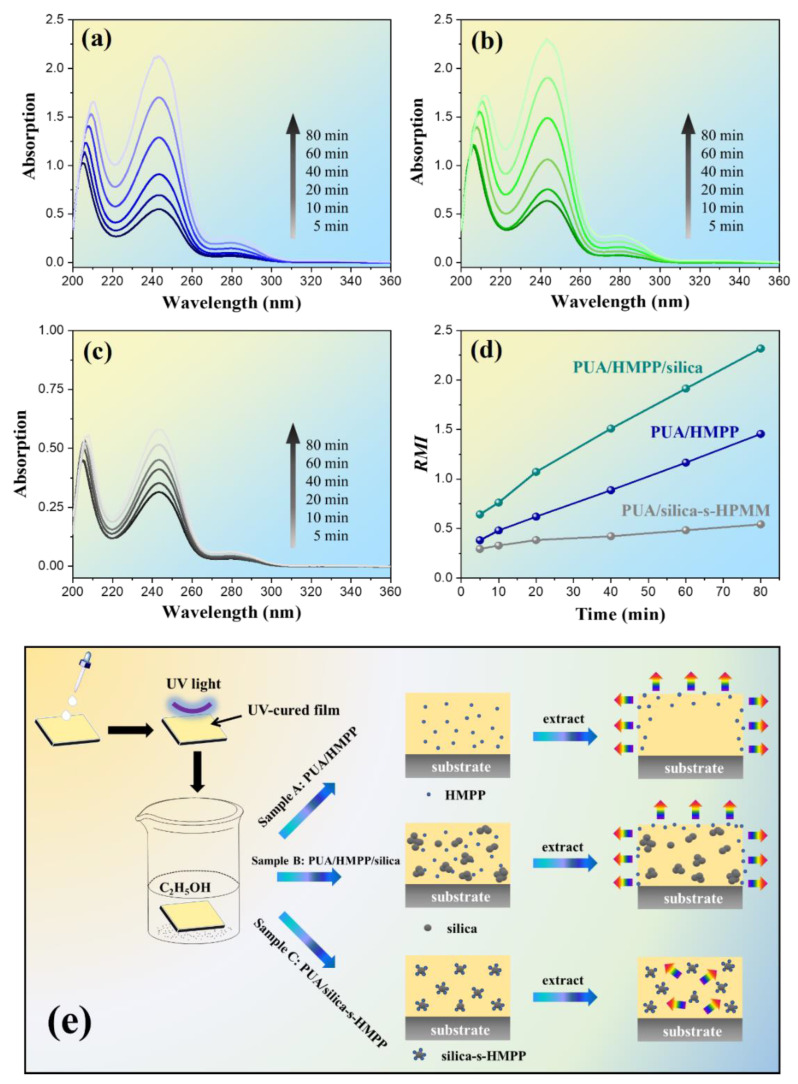
UV-vis absorption spectra of extractable content from UV-cured films (**a**) PUA/HMPP, (**b**) PUA/HMPP/silica, (**c**) PUA/silica-s-HMPP, (**d**) relative migration index (RMI) vs. immersion time, and (**e**) test method and mechanism of migration of different photoinitiators in the UV-cured films.

Through the chemical reaction to anchor the photoinitiator HMPP onto the nanoparticle surface, there can be a resultant decreased mobility of residual PIs in the UV resin matrix. Therefore, absolute ethanol was used to extract the residual free photoinitiator from the UV-cured samples, including PUA/HMPP, PUA/HMPP/silica, and PUA/silica-s-HMPP. After ethanol extraction, quantitative analysis by UV-vis absorption spectroscopy was conducted, and the results are shown in Figure 5. The schematic of the test and the mechanism of different photoinitiator migrations out from the UV-cured films is exhibited in Figure 5e. The UV-cured films were peeled off from the glass substrate and immersed in absolute ethanol solution at different times to record the UV-vis absorption spectra of the extraction solution at 5, 10, 20, 40, 60, and 80 min, respectively. With the same HMPP content, sample PUA/HMPP/silica shows an absorption spectra of extractable residual PIs a little stronger than PUA/HMPP all the time. As expected, sample PUA/silica-s-HMPP initiated by silica-s-HMPP displays absorption spectra much less than the other samples. It indicates that the inorganic nanoparticles may damage the network regularity of the matrix and prompt the removal of HMPP from the elastomer matrix. However, the inorganic and organic hybrid photoinitiator silica-s-HMPP is an efficient method for HMPP to exist stably in the UV film through immobilizing HMMP on the silica surface.

The Beer-Lambert Law states there exists a significant linear relationship between the concentration and absorbance of the solute. The relative migration index of photoinitiator (RMI) at different time of UV-cured film was calculated by the following Equation (2):

(2) $${\mathrm{RMI}=A}_{t}{/A}_{0}\;$$

where *A*_0_ is the absorbance at 243 nm of HMPP, silica-s-HMPP, and a mixture of HMPP and silica, which is from Figure 2d and *A*_t_ is the absorbance at 243 nm with a certain immersion time. RMI illustrates the relative migration index of the photoinitiator in the UV-cured film. As shown in Figure 5d, the hybrid silica-s-HMPP displays the lowest RMI, has a significant immobilization in the UV-curing film, and is better than that of the commercial photoinitiator HMPP and the mixture of HMPP and silica. The immobility of silica-s-HMPP may be due to two factors: the stabilized chemical linkage between silica and HMPP and the strong interfacial interaction between the photoinitiator and the resin matrix. For the PUA/silica-s-HMPP, the hybrid silica-s-HMPP is efficiently immobilized in the film matrix with a slight migration effect.

### 3.5. 3D Printing Resolution and Printed Objects from the PUA/Silica-s-HMPP Ink

**Figure 6:**
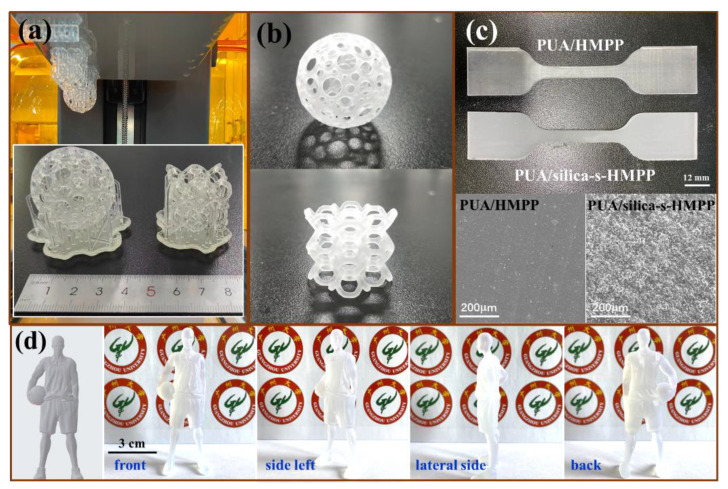
Digital photos of 3D printed objects (PUA/silica-s-HMPP system) acquired (**a**) during the printing process, and (**b**) after cleaning in isopropanol firstly and then post-cured by UV-irradiation. (**c**) Digital photo and SEM images of the surface finish of the PUA/HMPP and the PUA/silica-s-HMPP system 3D printed objects. (**d**) A 3D model and digital photos of 3D printed object of Sakuragi Hanamichi standing in different directions (printed by the photosensitive resin of the PUA/silica-s-HMPP system), respectively.

The digital photos of 3D printed objects of the PUA/silica-s-HMPP system acquired during the printing process are exhibited in Figure 6a. Furthermore, stent-free printed objects are cleaned in isopropanol first to remove the unreacted residual resin and then post-cured by UV-irradiation, as shown in Figure 6b. The 3D prints exhibit excellent printing resolution and an opaque appearance after cleaning with isopropanol. Digital photos of 3D printed dumbbell sharp objects of PUA/HMPP and PUA/silica-s-HMPP and the SEM images of these sample surfaces are shown in Figure 6c. PUA/HMPP appears more transparent than PUA/silica-s-HMPP due to the refraction of light by silica particles. The SEM image of PUA/HMPP reveals that the thickness of fabricated layer on the object surface is approximately 102.1 μm, indicating that the printed object presents a high resolution with a smooth surface. Even the rough-surface of PUA/silica-s-HPMM shown in th eSEM image reveals uniformly dispersed nanoparticles in the resin matrix in our preferred orientation and arrangement (layer-by-layer) with a thickness of 104 μm. We further applied the silica-s-HMPP as the photoinitiator for the 3D printing of Sakuragi Hanamichi, a basketball player in the animation, and the 3D model and digital photos from different angles are shown in Figure 6d. We discovered that the hybrid photoinitiator silica-s-HMPP can improve the printing accuracy to a certain extent by ensuring homogeneous dispersion in the PUA matrix and the prevention of light leakage from the required light exposure area due to the optical opacity of silica nanoparticles. Based on the above analysis, the novel hybrid photoinitiator of silica-s-HMPP added into the PUA matrix endows the printed objects with excellent mechanical properties and high resolution, and allows for a prominent improvement in migration resistance. These combinations have the potential to be used in novel 3D printing material directions.

## 4. Conclusions

In summary, we investigated the impacts of different kinds of photoinitiators including commercial HMPP and novel hybrid photoinitiator silica-s-HMPP on the structure and comprehensive performance, especially the anti-migration performance of organic and inorganic hybrid PUA elastomer. The results demonstrated that the inorganic-organic photoinitiator silica-s-HMPP particles are more thermal stable than the original photoinitiator HMPP and can be applied in SLA 3D printing. Compared to both the unfilled and filled UV-cured samples, the PUA/silica-s-HMPP system can effectively improve the filler dispersion in the matrix and possesses the highest mechanical properties such as tensile strength and successful 3D printing. Meanwhile, due to the multifunctional properties of silica-s-HMPP, the UV-cured sample had a lower content of extractable residual photoinitiators which led to the excellent stability of fragments of photoinitiator. Considering the lower photoinitiating activity of silica-s-HMPP, our future work will focus on the improvement of the photopolymerization rate.

## Figures and Tables

**Table 1:** Formulas of the UV-cured hybrid materials.

Sample	RJ-426	RJ-425	ACMO	HMPP	Silica	Silica-s-HMPP
PUA/HMPP	60	40	5	0.575	0	0
PUA/HMPP/silica	60	40	5	0.575	4.094	0
PUA/silica-s-HMPP	60	40	5	0	0	6

## Data Availability

Not applicable.
